# Toxic Effects of Low-Level Long-Term Inhalation Exposures of Rats to Nickel Oxide Nanoparticles

**DOI:** 10.3390/ijms20071778

**Published:** 2019-04-10

**Authors:** Marina P. Sutunkova, Svetlana N. Solovyeva, Ilzira A. Minigalieva, Vladimir B. Gurvich, Irene E. Valamina, Oleg H. Makeyev, Vladimir Ya. Shur, Ekaterina V. Shishkina, Ilya V. Zubarev, Renata R. Saatkhudinova, Svetlana V. Klinova, Anastasia E. Tsaregorodtseva, Artem V. Korotkov, Eugene A. Shuman, Larisa I. Privalova, Boris A. Katsnelson

**Affiliations:** 1 The Medical Research Center for Prophylaxis and Health Protection in Industrial Workers, 30 Popov Str., 620014 Ekaterinburg, Russia; marinasutunkova@yandex.ru (M.P.S.); solovyevasn@ymrc.ru (S.N.S.); ilzira@ymrc.ru (I.A.M.); gurvich@ymrc.ru (V.B.G.); sahautdinova@ymrc.ru (R.R.S.); klinovasv@ymrc.ru (S.V.K.); privalova@ymrc.ru (L.I.P.); 2 The Ural State Medical University, 17 Klyuchevskaya Str., 620109 Ekaterinburg, Russia; ivalamina@mail.ru (I.E.V.); larim@mail.ru (O.H.M.); Tsaregorodtseva@mail.ru (A.E.T.); cardiovektor.inbox@gmail.com (A.V.K.); evgenyshuman@gmail.com (E.A.S.); 3 The Institute of Natural Sciences, the Ural Federal University, 620000 Ekaterinburg, Russia; vladimir.shur@urfu.ru (V.Y.S.); ekaterina.shishkina@urfu.ru (E.V.S.); i.v.zubarev@urfu.ru (I.V.Z.)

**Keywords:** nickel oxide nanoparticles, inhalation exposure, pulmonary responses, systemic toxicity, genotoxicity, bioprotectors

## Abstract

Rats were exposed to nickel oxide nanoparticles (NiO-NP) inhalation at 0.23 ± 0.01 mg/m^3^ for 4 h a day 5 times a week for up to 10 months. The rat organism responded to this impact with changes in cytological and some biochemical characteristics of the bronchoalveolar lavage fluid along with a paradoxically little pronounced pulmonary pathology associated with a rather low chronic retention of nanoparticles in the lungs. There were various manifestations of systemic toxicity, including damage to the liver and kidneys; a likely allergic syndrome as indicated by some cytological signs; transient stimulation of erythropoiesis; and penetration of nickel into the brain from the nasal mucous membrane along the olfactory pathway. Against a picture of mild to moderate chronic toxicity of nickel, its in vivo genotoxic effect assessed by the degree of DNA fragmentation in nucleated blood cells (the RAPD test) was pronounced, tending to increasing with the length of the exposure period. When rats were given orally, in parallel with the toxic exposure, a set of innocuous substances with differing mechanisms of expected bioprotective action, the genotoxic effect of NiO-NPs was found to be substantially attenuated.

## 1. Introduction

Like many other metal-oxide nanoparticles species that we toxicologically characterized in experiments during 2009–2017 and reported in research papers and overviews [1,2], nickel-oxide nanoparticles (NiO-NPs) are of special interest for industrial toxicology and occupational health risk assessment and management. Not only are engineered NiO-NPs produced for various industrial applications (such as photoelectric and recording materials, catalysts, sensors, and ceramics), but they also make up a substantial proportion in the particle size distribution of condensation aerosols generated by traditional metallurgical and arc-welding technologies. While comprising chemically similar fine micrometer and submicron-sized (>100 nm) particles, such aerosols commonly contain the conventional nanoscale fraction (<100 nm) as well [1].

It is no wonder that NiO-NP toxicology has been, and still is, the subject-matter of numerous experimental studies. However, the prevailing majority of the relevant studies were done in vitro on cultured cells (e.g., [3,4,5,6,7]) or sometimes in vivo on daphnia [8] and drosophila [9], while the experiments on laboratory rodents used mostly single-shot or repeated intratracheal instillations [7,10,11,12,13,14,15,16] and, occasionally, oral administration [17].

We have not come across any studies that would examine the chronic toxicity of NiO-NPs by a reasonably large number of informative indicators using a long-term inhalation exposure. In the studies we know of, such exposure lasted no longer than 4 weeks [18,19,20,21].

In summary, the above-mentioned and similar publications have demonstrated pronounced toxicity of NiO-NPs on cell, organ, and system (mainly respiratory) levels and moderate in vivo genotoxicity. They have also shown that one of the primary mechanisms of action on the cell is the generation of free oxygen radicals. All of these phenomena are, to a greater or lesser degree, characteristic of any metal and metal-oxide nanoparticles (MeO-NPs) studied so far. However, toxicologists still lack enough quantitative or even descriptive data to support a reliable assessment of health risks associated with occupational and environmental exposures to NiO-NPs. 

In particular, although Ni as a chemical element is a recognized human carcinogen due to its genotoxicity and although the genotoxic effects of NiO-NPs have been demonstrated not only on cell lines or on drosophila [9] but also in some acute (for instance, [16]) or even subchronic [22] experiments on rats, no such data seem to be available from chronic inhalation studies, nor is there any reliable assessment of pulmonary and systemic non-genotoxic adverse effects. One therefore cannot be certain that it is the genotoxicity of NiO-NPs in actual industrial and residential conditions that is to be regarded as an adverse characteristic of top priority. 

Last but not least, virtually nobody outside our team in nano-toxicological research has ever tried to raise the problem of enhancing the organism’s resistance to the systemic toxic action and genotoxicity of the MeO-NP species under consideration or of any other such NPs. Although we developed [22] a combination of bioprotectors and successfully tested it against combined nickel-manganese NiO-NP + Mn_3_O_4_-NP intoxications [23], it was still unknown whether such a combination was effective against the toxicity of NiO-NPs proper. Moreover, despite our wide experience in bioprotection against acute and subchronic effects of various metals in the form of ions or nanoparticles [22,24], we have not yet performed such studies for chronic inhalation nano-intoxications.

## 2. Results and Discussion

### 2.1. A Pilot Experiment

As was emphasized in the introduction, the experimental data on the inhalation toxicity of NiO-NPs reported so far have been obtained [18,19,20,21] for exposures that were not long-term and in this respect not comparable, even for the short lifespan of rats, with possible occupational exposure durations that are of primary interest in the area of preventive industrial toxicology. Indeed, if we assume that the average lifespan of a laboratory rat is equal to 30 months, inhalation experiments that last 4 weeks (i.e., only about 3% of the total lifespan) are negligibly short in comparison with a human working lifetime (which, e.g., in Russia amounts, even under hazardous occupational conditions, to ~50% of the standard human lifetime). Moreover, a tolerable single four-hour exposure of rats in “nose only” inhalation setups is much shorter than the working day, even if it is reduced under hazardous conditions. This circumstance further shortens the total chronic exposure in experiments compared with actual occupational contexts.

For these reasons, we designed a chronic inhalation experiment intended to last 3–10 months and involving an average concentration of 1.0 mg/m^3^ of NiO-NPs in the air inhaled by rats. This exposure concentration was considered to be moderate and to allow for the position of regulatory toxicology in relation to metallic nickel and its water-insoluble compounds in workroom air (for example, the US OSHA established a PEL equal just to 1.0 mg/m^3^ as a TWA concentration). Assuming that any chemical compound of nickel is likely to be more toxic compared with its iron-containing counterpart, we expected that, for nano-sized nickel-oxide particles, this concentration would be even more toxicologically effective (rather than being innocuous) than for Fe_2_O_3_ iron oxide nanoparticles in about the same concentration (1.14 ± 0.01 mg/m^3^) tested by us previously in a similar inhalation experiment [25]. At the same time, we assumed that the contrary role as a factor that determines the comparative toxicity of these two metal-oxide nanoparticle species would be the nearly twice as great diameter of NiO-NPs (23 ± 5 nm) in comparison to Fe_2_O_3_-NPs (14 ± 4 nm). 

Nevertheless, the expectation of a substantial toxicity of nickel-oxide nanoparticles in this concentration unexpectedly came not only true but was even exceeded so much that already after the first 1–2 exposures the clinical picture of an acute intoxication became evident. We were afraid, therefore, that the animals would not live long enough for a chronic intoxication to develop in them. We thus decided to interrupt the experiment after only five daily exposures and to begin a new one with the average concentration of NiO-NPs lowered 4–5 times (see Section 3).

However, we have measured in these rats at least some of the indices that were planned by the design of the chronic experiment (see Section 3) in order to use them as predictors of the results in the longer-term experiment. Therefore, we regard the first inhalation experiment as a pilot rather than a failure.

As follows from Table 1 (which presents mostly those indices for which the exposed group differed from the controls statistically significantly), even though the total exposure period was short, certain functional effects of toxic action did manifest themselves. We noted the following:

- an increase in liver mass typical of subchronic and chronic intoxications with practically all metal-oxide nanoparticles ever studied by us;
- elevated release of lactate dehydrogenase in the blood due, probably, to the toxic damage to the liver cells and to the pulmonary macrophages;
- leukocytosis;
- systemic inhibition of the oxidation-reduction energy metabolism, the integrated cytochemical indicator of which is the suppression of succinate dehydrogenase activity in blood lymphocytes, which we repeatedly observed in experiments involving practically any of the toxic metals in any form [23,26,27,28];
- enhanced lipid peroxidation judging by the increased concentration of malondialdehyde in the blood;
- stimulation of erythropoiesis suggested by an increase in the erythrocyte count (along with an increased proportion of reticulocytes), hematocrit, and hemoglobin content of the blood.

Such a quasi-beneficial hematological effect is astonishing not only because various metal-induced intoxications go along with this or because of the degree of anemia with different mechanisms of development but also because intraperitoneal subchronic intoxication caused by similar NiO-NPs was previously found by us [23] to bring about just such anemia. We will consider this apparent contradiction below (Section 2.2) when discussing the findings of the main chronic inhalation experiment, in which such stimulation was again observed. As for the pilot experiment being considered, it is noteworthy that even one week after its termination the red blood still displayed a response, although it was less pronounced. For instance, the proportion of reticulocytes was equal to 25.86 ± 3.88‰ against 15.22 ± 1.37‰ in the control group (*p* < 0.05).

In 24 h after the last exposure, a dramatic increase in both the bronchoalveolar lavage fluid (BALF) total cell count and the number of alveolar macrophages (AMs) and neutrophil leukocytes (NLs) in comparison with the corresponding indices in the control group was observed (Table 2). The NL/AM ratio was, however, somewhat decreased, which is not characteristic of the immediate pulmonary response to the deposition of any particles and, in particular, of highly cytotoxic ones [1,23,26,27,28,29,30,31,32]. Three weeks later, the differences between the NiO-NP-exposed and control groups in the BALF total cell count and the AM numbers remained statistically significant although somewhat decreased, while the initial neutrophilic response disappeared.

Recently we published (for the first time in nanotoxicology, as far as we know) a cytological analysis of tissue imprints from organs of rats exposed subchronically to intraperitoneally injected metal-oxide nanoparticles in experiments with Al_2_O_3_-NPs, TiO_2_-NPs, and SiO_2_-NPs administered separately or in different combinations [27]. That analysis proved to be rather informative. Simultaneously we explored the potentialities of this approach in the pilot inhalation experiment under discussion now and obtained some interesting results (Table 3). 

In particular, the following findings are noteworthy:

- the lungs had increased proportions of explicitly degenerated alveolar macrophages and of eosinophils;
- the liver had increased proportions of explicitly degenerated hepatocytes, activated Kupffer’s cells, neutrophils, and eosinophils;
- the kidneys had increased proportions of explicitly degenerated cells of proximal (and to a much lesser degree, distal) convoluted tubules;
- the spleen displayed a decrease in the proportion of mature lymphocytes together with prolymphocytes and a respective increase in the proportion of other white cells, especially eosinophils.

Thus, notwithstanding rather a short inhalation period and a seemingly low exposure level, we can see some typical adverse effects on target organs along with hyperergic toxic inflammation evidenced by a marked eosinophilic response. It would be an enticing hypothesis to connect the latter with the well-known systemic nickel allergy syndrome if a similar response had not been observed in the above-mentioned experiments with three other NPs of elements, none of which is, unlike nickel, a recognized potent sensitizer [27]. In this respect, we should mention the urgency of the well-recognized general problem of “nanoparticles and allergy” [33], which has not yet been solved satisfactorily. 

### 2.2. A Long-Term Low Level Exposure

As will be described in detail in Section 3, in this experiment, the NiO-NP concentrations were approximately four times lower compared with the pilot one (0.23 ± 0.01 mg/m^3^ instead of 1.0 ± 0.1 mg/m^3^). In other respects the mode and duration of weekly exposures were the same (4 h a day, 5 times a week), but the total exposure period was prolonged up to 3, 6, or 10 months.

As follows from the comparison of the data presented in Table 2 and Table 4, the shifts in the cytological characteristics of the BALF 24 h after the final inhalation exposure to nanoparticles in the chronic inhalation experiment were, unlike the pilot one, of the usual character (an increase in total cell count due to the recruitment of both AMs and NLs with an increased NL/AM ratio in comparison with the control group). Believing that such shifts were determined mainly by the response to the last portions of particles deposited in the lower airways, we did not expect them to increase by increasing the overall duration of the exposure period. Indeed, the cytological characteristic of the BALF after the 6-month and 10-month exposure was in principle the same. However, over both of these experimental periods, the total cell counts and the AM and NL numbers were much lower than after the 3-month exposure. 

Our studies demonstrated long ago that the intensity of the protective compensatory recruitment of alveolar macrophages and, particularly, neutrophil leukocytes into the lower airways is higher as the particles become more cytotoxic and thus as the mass of produced macrophage breakdown products (MBPs) regulating this recruitment increases [29,34]. However, the recruitment of new echelons of phagocytizing cells in response to an intratracheal instillation of one and the same dose of sterile MBPs (obtained by freezing-thawing or ultrasonication of a macrophage culture in aseptic conditions) greatly depends on the total reactivity of the organism, which may change under the effect of various factors [31]. Assumingly, in the current experiment, a similar change in the reactivity of the organism took place. This change might be, on the one hand, a result of the chronic intoxication suppressing this reactivity and, on the other hand, a manifestation of the organism’s partial adaptation to the impact of inhaled particles.

At the same time, the increased activity of the BALF supernatant’s enzymes (Table 5) is likely to reflect the destruction of cells on the free surface of the lungs’ pulmonary region under the impact of cytotoxic particles. Since the exposure to NiO-NPs was constant, it is not surprising that these biochemical indices were relatively stable in all three experimental periods.

In Table 6 we present all functional indices for the organism’s status measured in both exposed and control (sham-exposed) rats to find whether any of them revealed statistically significant and/or consistent shifts that might be attributed to the NiO-NP toxic action.

In fact, like the above-discussed cytological BALF indices, the systemic toxic action effects of inhaled nanoparticles presented in this table suggest that the rat organism was gradually adapting to some extent to the low-level inhalation exposure. In this respect, of particular interest are the red blood indices. Previously we found [23] that under a subchronic intraperitoneal exposure to the NiO-NPs they generally developed anemia. At the same time, under low-level inhalation burdens we can see signs of erythropoiesis stimulation both in the short-term pilot experiment (see Section 2.1) and by the end of the 3rd month in the main experiment. In the latter case, these signs included an elevated hemoglobin content, an increased erythrocyte count with an elevated proportion of reticulocytes, and an elevated hematocrit. However, in the subsequent terms of this investigation, the possible reaction of the marrow bone was evidenced only by a statistically significant increase in the proportion of reticulocytes. 

The effect produced by various chemical forms of nickel on erythropoiesis is not something new, although we have not come across any references to such nanoparticle action in this metal. Thus, Sunderman et al. [35] observed an enhanced generation of erythropoietin with an increase in hematocrit in guinea pigs and rats in response to intrarenal administration of nickel subsulfide. On the contrary, anemia is known to develop in rats under intoxication, for example, with nickel chloride [36]. Phase-related changes in the red blood indices under a 4-month (every other day) inhalation exposure to micrometer particles of metallic nickel or nickel oxide at a concentration of 350 mg/m^3^ were described a long time ago [37]. An enhancement of erythropoiesis was noted in 2 weeks, lasted for 1.5 months, and then decreased to the initial level or fell below it. Later on, a study involving continuous 3-month inhalation exposure to a rather coarse metallic nickel dust at a concentration of 0.5, 0.1, and 0.02 mg/m^3^ also revealed an increase in the erythrocyte count in the peripheral blood after 2.5 months of exposure followed by normalization of this index by the end of the exposure period [38].

It is interesting that we found a similar alternating pattern of response to chronic inhalation of NiO-NPs for such an important toxic effect as energy metabolism suppression as well. Indeed, the typical decrease in the cytochemical index of SDH activity in blood lymphocytes was pronounced and statistically significant by the end of the 3rd month but was absent in the next month. On the contrary, the intensity of lipid peroxidation estimated by the concentration of malondialdehyde (MDA) in the blood serum increased significantly only towards the end of the long-term exposure period. Meantime, it was significantly reduced in the first term (which is an extremely rare and hard-to-explain effect) and virtually unchanged in the mid-term. No significant changes in reduced glutathione and total SH-groups in the blood-typical effects for different metal intoxications—were observed in this instance. 

**Figure 1:**
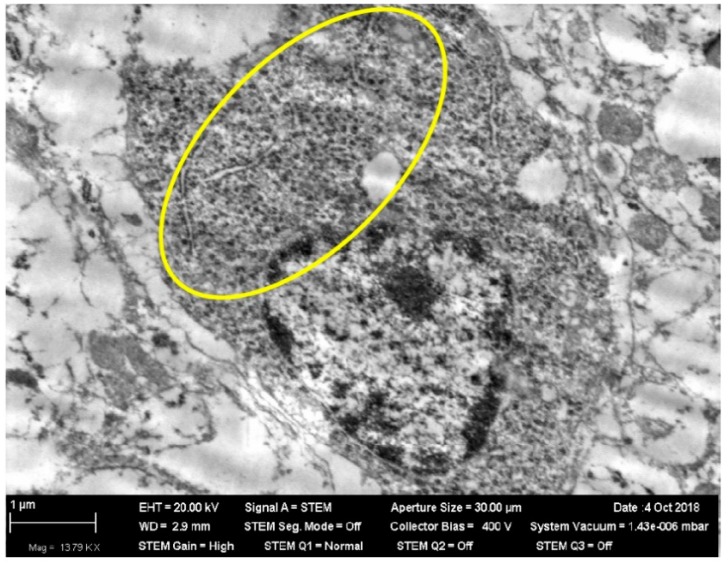
Nanoparticles in the body of a neuron in an olfactory bulb of rat brain after 3 months of inhalation exposure to NIO-NPs at a concentration of 0.23 mg/m^3^. STEM; magnification: 13,790×. (See within the yellow oval.)

**Figure 2:**
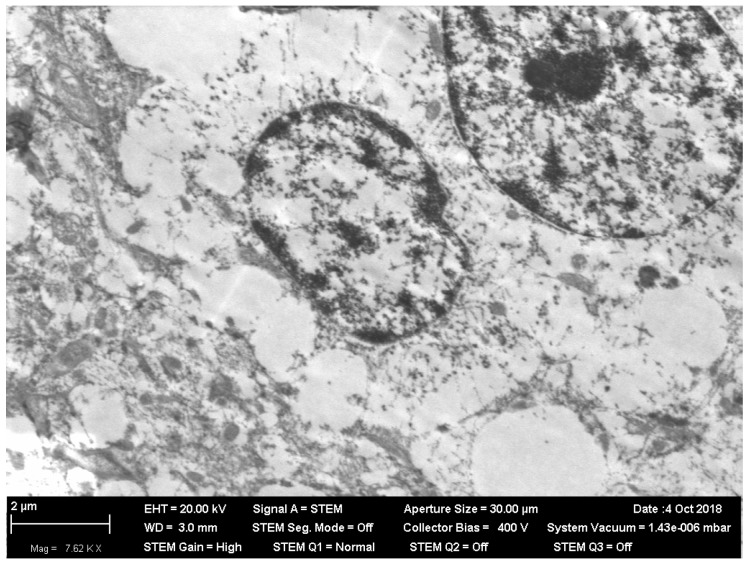
Autolysis in the body of a neuron in an olfactory bulb of rat brain after 3 months of inhalation exposure to NIO-NPs at a concentration of 0.23 mg/m^3^. STEM; magnification: 7620×.

**Figure 3:**
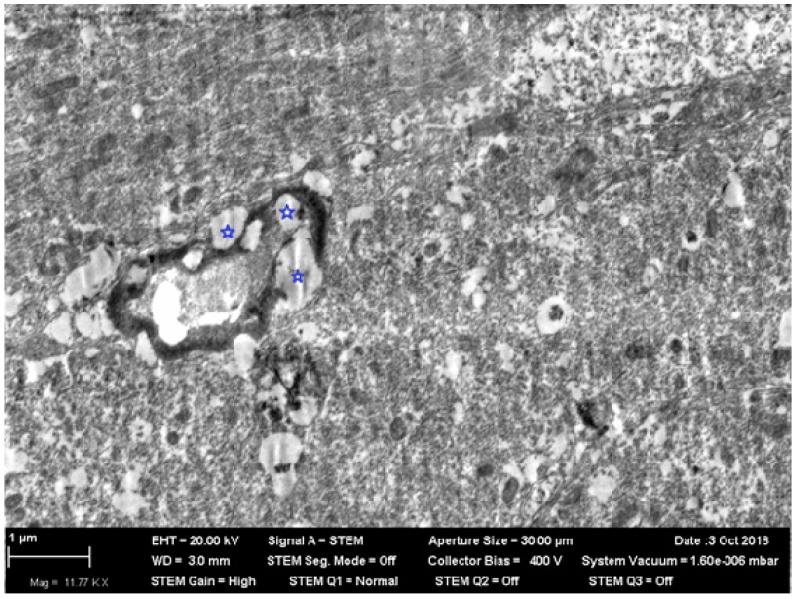
Demyelinized sites (noted with asterisks) on the fibers of an olfactory bulb of rat brain after 3 months of inhalation exposure to NiO-NPs at a concentration of 0.23 mg/m^3^. STEM; magnification: 11,770×.

What is particularly noticeable is the insignificance and inconstancy of many other (including organ-specific) shifts caused by low-level chronic inhalation exposure to NiO-NPs. Thus, none of the usually observed signs of increased inhibition processes in the CNS (longer index of summation of subthreshold impulses, suppression of the behavioral indices of exploratory, and general motor activities) displayed, in the chronic inhalation experiment, any statistically significant difference from the control value. Rather, they generally tended to be oppositely directed, especially at the end of the exposure period. This circumstance is particularly interesting because under inhalation exposure one of the targets for nanoparticles initially deposited in the nasal ways is the brain, to which they move along the fibers of the olfactory nerve [25,39,40,41]. Indeed, the electron microscopy of olfactory bulb preparations from the rat brain in this experiment revealed both nanoparticle retention (Figure 1) and ultrastructural damage (Figure 2 and Figure 3). The averaged nickel content of the homogenized tissues of the entire brain was just slightly increased, which indirectly confirms the local nature of NiO-NP retention limited to the olfactory tract.

There was no noticeable change in the relative mass of the liver, spleen, and kidneys or in indices of liver functioning (the protein and separate protein fractions content in the blood serum and aminotransferase activities) or of kidney functioning (diuresis, urine density, and endogenous creatinine clearance). At the same time, in all three periods of this inhalation exposure, increased bilirubin content in the blood serum and proteinuria after 6 and 10 months of exposure (statistically significant only by the 6th month) was observed.

The low manifestation of the hepato- and nephrotoxicity effects may be explained by the fact that the nickel content of these organs, both total (by the AES data) and as NiO (by EPR-spectroscopy data), virtually did not differ from the control values. Thus, by the end of the 10-month period, for the liver it was equal in the control group to 0.65 ± 0.22 and 0.06 ± 0.02 μg per gram of dry tissue, respectively, while in the exposed group it was equal to 0.73 ± 0.17 and 0.07 ± 0.03 μg/g; for kidneys, it was equal to 0.31 ± 0.08 and 0.03 ± 0.01 μg/g in the control group and 0.35 ± 0.06 and 0.02 ± 0.01 μg/g in the exposed group. No additional accumulation of nickel in the spleen of the exposed rats was observed: 0.25 ± 0.07 μg/g by the AES data and 0.01 ± 0.01 μg/g by the EPR data (against 0.27 ± 0.03 and 0.02 ± 0.00 μg/g in the control group). Meanwhile, both indices of blood nickel content in the exposed rats (0.48 ± 0.05 and 0.53 ± 0.14 μg/g) were higher than the corresponding control values (0.39 ± 0.07 and 0.30 ± 0.07 μg/g), but not by much, and the increase was statistically insignificant. 

**Figure 4:**
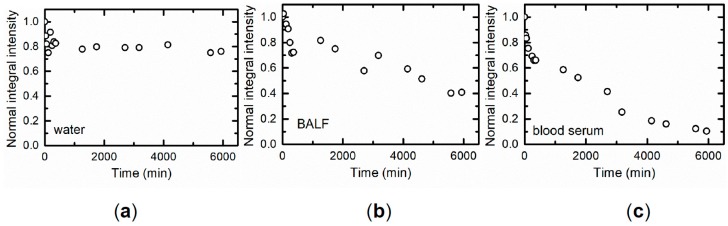
Decay kinetics of the Ni^2+^ EPR signal from a filter on which NiO-NPs deposited from the inhalation exposure chamber exhaust air (**a**) in de-ionized water, (**b**) in rat BALF supernatant, and (**c**) in sterile bovine blood serum.

A low chronic accumulation of NiO-NPs in the above-mentioned organs as well as in the lungs (see below) is presumably due mostly to its solubilization in biological milieus, which could be directly proved in vitro (Figure 4).

**Figure 5:**
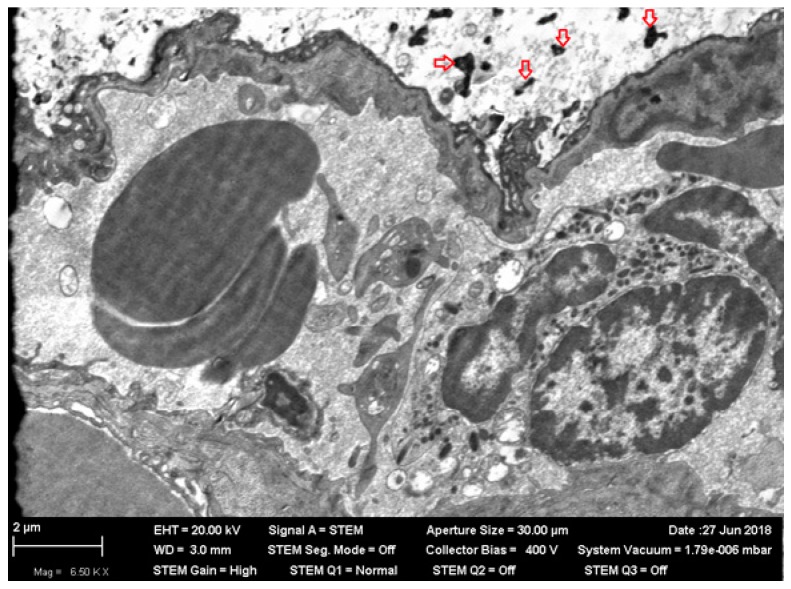
Rat lungs after 3 months of inhalation exposure to NiO-NPs at a concentration of 0.23 mg/m^3^. STEM; magnification: 6500×. Nanoparticle aggregates in the alveolar space are noted with arrows.

It is worth mentioning that in the case of inhalation exposure there is an increase in dry mass of lungs, a typical observation for any experimental pneumoconiosis. In this case, however, this increase grew statistically significant only by the 10th month. By this time, the retention of nickel in the lung tissue was the highest according to both the AES data (3.69 ± 0.35 against 0.96 ± 0.30 μg/kg in the sham-exposed group; *p* < 0.05) and the EPR spectroscopy (0.39 ± 0.04 and 0.09 ± 0.03 μg/kg, respectively; *p* < 0.05). Nevertheless, electron microscopy of the lungs (Figure 5) revealed numerous nanoparticles or their nano-sized aggregates in all periods of the study not only as internalized by phagocytic cells but also as located freely inside alveoli, which seems to be a result of their recent deposition from the inhaled air. 

**Figure 6:**
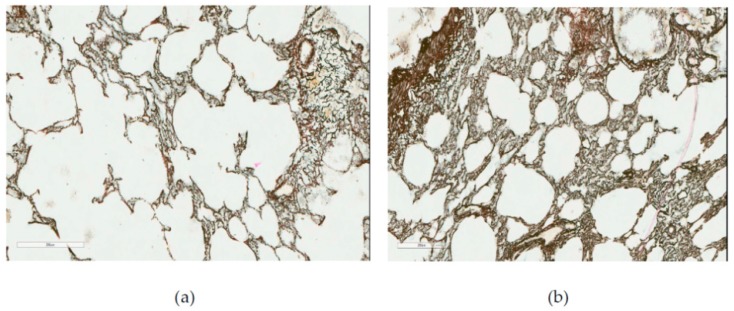
Rat lungs (**a**) after 10-month inhalation exposure to NiO-NPs and (**b**) in the control group of the same exposure period. Gomori’s silver impregnation, magnification: 400×. See description in the text.

At the same time, a histological investigation of the lung tissue by optical microscopy did not reveal, even towards the end of the experiment, any cellular fibrotic nodules typical of experimental pneumoconioses, or any thickening of interalveolar septae together with their diffuse fibrosis. On the contrary, the septae became thinner or were even destroyed (emphysema), while the reticulin framework was no coarser than in the control rats (Figure 6).

Besides the above-considered functional indices, all internal organs displayed appreciable, even if moderate, pathological changes in the histological structure confirmed by optical microscopy-based morphometric assessment (Table 7 and Table 8). The fact that there was no increase in the proportion of akaryotic hepatocytes after the 3-month exposure is quite consistent with the observation that in this period the retention of nickel in the liver was still negligibly low (according to AES data, 0.10 ± 0.05 against 0.08 ± 0.03 mg/kg in the control group; according to EPR, 0.02 ± 0.02 mg/kg and 0.00, respectively). Thus, the hepatotoxic effect, too, did not display any signs of the organism’s adaptation to the effects of NiO-NPs.

The reduced planimetric ratio of red pulp to white pulp in the spleen usually observed by us in subchronic intraperitoneal experiments with nanoparticles [1] was found in this inhalation experiment only by the first and last terms of investigation, and this ratio decrease was not statistically significant. However, the response of the white pulp is evidenced by an increase in the average diameter of lymphoid follicles observed in all periods of the experiment. Assumingly, it was a reactive follicular hyperplasia associated not as much with the retention of nanoparticles in this organ as with the probability of an allergic syndrome mentioned above (in the discussion of the data of Table 3).

**Figure 7:**
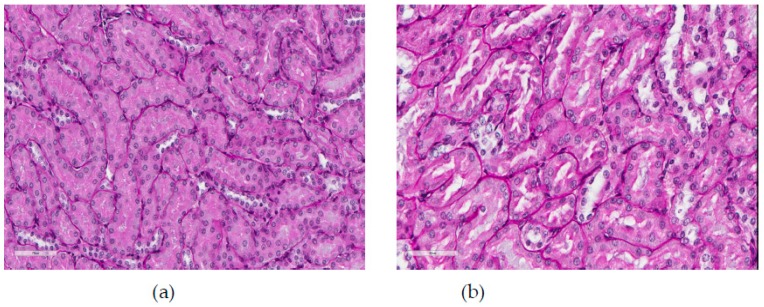
(**a**) Kidney of a control rat (proximal convoluted tubules with an intact brush border). (**b**) Kidney of a rat exposed to NiO-NP inhalation during 10 months (marked degenerative and necrobiotic changes in tubular epithelial cells up to their disappearance; partial destruction of the brush border). Periodic acid–Schiff (PAS) stain; magnification: 400×.

For kidneys, as is common [1], the most typical adverse effect of NiO-NP toxicity was damage to the tubular epithelium (Figure 7 and Table 8). The most obvious signs of such damage are brush border loss in proximal convoluted tubules and full epithelial desquamation. However, although the first of these effects was observed in all terms of the long-term inhalation exposure, it was statistically significant only in the 3-month term. The second of the named effects was statistically significant only in the 10-month term. For this term we only obtained a statistically significant difference in the average diameter of the glomeruli between the exposed and sham-exposed groups. It is easy to see, though, that this indicator in the exposed group was actually the same as in the previous periods, while in the control group it increased unexpectedly. We therefore abstain from its toxicological interpretation.

The cytological changes in tissue imprints (Table 9) were generally of the same character as in the pilot experiment (Table 3). At the same time, it is interesting that for some important indices of this kind (for example, the percentage of eosinophils in the liver and spleen imprints), we can see an attenuation of shifts caused by exposure as the duration of exposure increases, although in some cases (in particular, the percentage of degeneratively changed cells in liver and kidney imprints), such attenuation was not observed from the above morphometric characteristics of the histological preparations.

### 2.3. Genotoxic Effect

The most important effect of toxic exposure to NiO-NPs that we found in all three periods of inhalation exposure is a statistically significant increase (compared with the control values) in the genomic DNA fragmentation coefficient (C_fr_) in circulating nucleated blood cells (Table 6, the lowest line). Previously we discovered this effect, along with a similar increase in C_fr,_ in cells of marrow bone, the liver, and other organs, for the subchronic action of all metal-oxide nanoparticles that we studied [1]. It can be regarded as a reliable quantitative indicator of genotoxicity inherent in all of them in vivo. It is to be specially noted that, whereas in the sham-exposed group of this experiment the value of C_fr_ remained constant throughout the entire period of observation, in the exposed group it tended to increase as the duration of the inhalation exposure increased (the difference between the first two periods being statistically significant). Thus, with regard to the genotoxic effect, no tendency towards adaption of the organism to the adverse action of NiO-NPs is observable; on the contrary, this effect is gradually increasing. 

Another piece of evidence supporting the association of the genotoxic effect with nickel-oxide exposure is its attenuation by the background oral administration of a bioprotective complex (BPC), which we tested for the first three months of the experiment. Whereas for rats exposed to nanoparticles only, C_fr_ in this period was equal to 0.4480 ± 0.0017, for a similar exposure against the background administration of the BPC, it was 0.4264 ± 0.0008 (*p* < 0.05). The administration of the BPC alone to sham-exposed rats did not affect the control value significantly in any way.

## 3. Materials and Methods

For modeling chronic intoxication development under low-level but long-term inhalation exposures to NiO nanoparticles, the experiments were carried out on outbred white female rats from our own breeding colony with an initial body weight of 150–220 g, with a minimum of 12 animals in exposed and control groups. Rats were housed in conventional conditions, breathed unfiltered air, and were fed standard balanced food. The experiments were planned and implemented in accordance with the “International guiding principles for biomedical research involving animals” developed by the Council for International Organizations of Medical Sciences (1985) and were approved by the Ethics Committee of the Ekaterinburg Medical Research Center Medical for Prophylaxis and Health Protection in Industrial Workers. 

**Figure 8:**
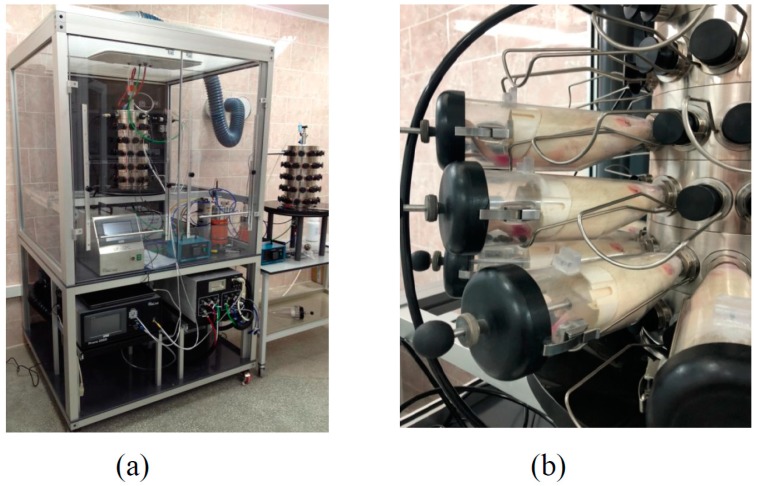
(**a**) The nose-only inhalation device (photographed without door wings of the draught cupboard) and, in the background, a similar tower for the sham exposure of the control group; (**b**) restrainers with rats attached to the exposure tower.

Airborne NiO-NPs were obtained by sparking from 99.99% pure nickel rods using the Palas DNP-3000 generator and fed into a nose-only exposure tower (CH Technologies, Westwood, NJ, USA) for 60 rats placed into individual restrainers (Figure 8). A device of the same design obtained from the same supplier was used for a sham exposure of control rats. 

**Figure 9:**
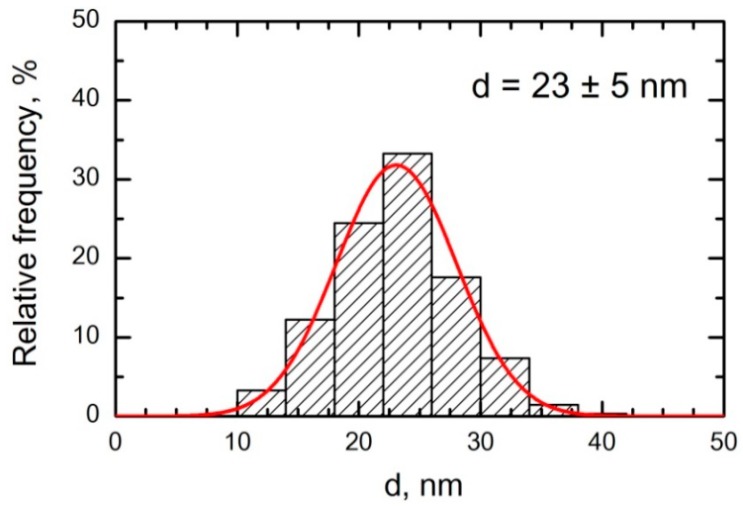
Particle or particle aggregate size distribution function obtained by statistical processing of 250 (N_0_) measured SEM NPs images of particles accumulated on a polycarbonate filter from the exposed rats’ breathing zone.

Particles collected on a polycarbonate filter and inspected under a scanning electron microscope (SEM) had a spherical shape and either were singlets or formed small aggregates. The latter, if compact, were measured as one particle. Even so, the particle size distribution (Figure 9) proved fairly clean-cut and restricted to the nanometric range with a mean (± s.d.) diameter of 23 ± 5 nm. The chemical identity of the NPs sampled on the filters was confirmed by Raman spectroscopy to be NiO. 

We studied NiO-NPs accumulated on the microporous PSI filter (Performance Systematix, Inc.—Grand Rapids, MI, USA) from the air exhausted from the exposure tower for testing particle dissolution in water, in normal saline, in cell-free BALF (broncho-alveolar lavage fluid) supernatant, or in sterile bovine blood serum. The NiO content was measured on frozen filters by the electron paramagnetic resonance spectroscopy (EPR) method using the Bruker EMXplus EPR Spectrometer (USA) with an operating microwave frequency of 9–10 Hz at a temperature of ~177 K. The first-order derivative of the absorption spectrum was recorded. The integrated intensity (the area under the absorption signal), which is proportional to the mass of NiO in the sample, was determined by double integration. To check how this mass reduced with exposure time in the fluid, the filter was extracted at preset intervals for EPR measurement and then put back into the vessel with the fluid being studied. 

Rats were exposed or sham-exposed for 4 h a day, 5 times a week, for 3, 6, or 10 months. Along with each single exposure, a sample of airborne nanoparticles was collected on an acetyl cellulose fine fiber filter attached to the inhalation device instead of a rat’s nose, while the volume velocity of air drawn through the filter was being monitored. The mass of Ni retained on it was determined with an atomic absorption spectrometer ContrAA 700 (Analytic Jena A, Jena, Germany) and translated into the mass of NiO and then into its air concentration as mg/m^3^. 

Twenty-four hours after the final exposure in each of these periods, we performed (a) a broncho-alveolar lavage to obtain a fluid (BALF) for cytological and biochemical characterization and (b) the following toxicometric tests:

1. weighing of the body;
2. estimation of the central nervous system’s ability to induce temporal summation of sub-threshold impulses—a variant of withdrawal reflex and its facilitation by repeated electrical stimulations in an intact, conscious rat;
3. recording of the number of head-dips into the holes of a hole-board (which is a simple but informative index of the exploratory activity frequently used for studying behavioral effects of toxicants and drugs) as well as of the number of squares crossed during the same time interval—as a measure of motion activity;
4. collection of daily urine for analysis of its output (diuresis), specific gravity (density), protein, total coproporphyrin, δ-aminolevulinic acid (δ-ALA), urea, uric acid, creatinine, and Ni content;
5. sampling of capillary blood from a notch on the tail for examining the hemogram and hemoglobin content and for cytochemical determination of succinate dehydrogenase (SDH) activity in lymphocytes (by the reduction of nitrotetrazolium violet to formazan, the number of granules of which in a cell was counted under immersion microscopy).

The rats were then sacrificed by semi-decapitation, and blood was collected by exsanguination. The liver, spleen, kidneys, and brain were weighed. The biochemical indices determined from the blood included reduced glutathione (GSH), total serum protein, albumin, globulin, bilirubin, ceruloplasmin, malondialdehyde (MDA), alkaline phosphatase, alanine- and asparate-transaminases (ALT, AST), catalase, gamma glutamyl transferase, SH-groups, urea, uric acid, creatinine, thyrotropic hormone of hypophysis, thyroxin, and triiodothyronine.

All the routine clinical laboratory tests on blood and urine were performed using well-known techniques described in many manuals (for instance, [42]).

The total nickel content of the liver, spleen, kidneys, and brain was determined by an atomic emission spectrometer with inductively coupled plasma, iCAP-6500 Duo (Thermo Scientific, Waltham, MA, USA). Samples of freeze-dried homogenized tissue were subjected to acid digestion with the help of a MARS 5 microwave accelerated reaction system. Due to the paramagnetic properties of nickel, the Ni^2+^ content of the same tissues was also studied using an electron paramagnetic resonance spectrometer, EMX Plus Bruker (Bruker, Germany). This method enabled us to measure the metal content in the chemical form of NiO (and, thus, as particulate Ni).

Liver, spleen, kidney, and brain tissue sections were prepared from 4 rats in each treated and control group for histological examination by hematoxilin-eosine staining and, where necessary, Periodic acid–Schiff (PAS), Nissl, or Perl’s stain. We used the Avtandilov’s planimetric ocular grid for morphometric characterization of the spleen. Liver and kidneys were characterized morphometrically using the programmed image-recognition system CellSens (Olympus, Ekaterinburg, Russia).

In addition, the pulmonary and brain accumulation of NPs and the ultrastructure of respective tissues were visualized by means of transmission electron microscopy (TEM). To this end, pieces of an organ were fixed in 2% paraformaldehyde and 2.5% glutaraldehyde in a cacodylate buffer with 5% sucrose at pH 7.3, post-fixed in 1% osmium tetroxide, contrasted with uranyl acetate *en bloc*, and embedded in epoxy resin (Spurr). This sample preparation procedure was carried out in a microwave tissue processor, HISTOS REM (Milestone, Milan, Italy). Semi-thin (900 nm thick) sections of epoxy blocks were stained in toluidine blue with the addition of 1% borax and examined under the optical microscope for choosing a site for TEM. The 60 nm ultrathin sections of this site obtained with the help an ultramicrotome (Power Tome, «RMC», Tucson, AZ, USA) were contrasted with uranyl acetate and lead citrate. Grid-mounted sections were investigated in an electron microscope, AURIGA («Carl Zeiss; MT», Oberkochen, Germany) in the STEM mode in the range of magnifications 1200–200000.

### 3.1. Testing of Genotoxicity (the Random Amplification of Polymorphic DNA (RAPD) Test.)

Having assessed the adverse effects of subchronic intoxication caused in rats by many metal and metal oxide nanoparticle species, we have proved that virtually all of them induce an increase in genomic DNA fragmentation in cells of different tissues, always including circulating nucleated cells of blood [1]. Therefore, the measuring of such fragmentation, albeit in these cells only, may serve as a relatively simple but sufficiently informative index (indeed a kind of mirror) of in vivo nanoparticle genotoxicity on the organism level.

The blood samples were collected in special vessels cooled to −80 °C. These were then promptly delivered in cryocontainers to a specialized genetic laboratory. 

To isolate DNA from the cells, we used a GenElute (Sigma) set of reagents in accordance with the manufacturer’s guidelines for use. The DNA content of the samples was determined spectrophotometrically (Ultraspec 1100 pro, Amersham Biosciences, Ltd., Amersham, UK), and they were then frozen and stored at −84 °C in a kelvinator (Sanyo Electric Co., Ltd., Moriguchi, Japan) until the beginning of the implementation of the RAPD (random amplified polymorphic DNA) test performed as in many previous experiments of ours (e.g., [27]). This technique allows one to define quantitatively the degree of DNA fragmentation as an estimate of a harmful agent’s genotoxicity and to reveal the respective attenuative effect of bioprotectors studied. The method is based on the following fact: unlike a fragmented DNA, which forms the so-called comet tail in the agarose gel electrophoresis, a non-fragmented DNA displays a very low degree of migration and virtually stays in the same place (comet head). The degree of DNA migration is directly related to the degree of DNA fragmentation. DNA amplification was carried out using specific primers and tritiated nucleotides. 

In contrast with the traditional comet assay, which is based on subjective visual estimation of the degree of damage to DNA of cells, the RAPD test allows for a quantitative determination of the degree of DNA fragmentation. In our experiment, the separation of DNA was done by the standard agarose gel-electrophoresis procedure. The polymerase chain reaction (PCR) was carried out using specific primers complementary to the Alu sequences and nucleotides (dCTP, dATP, and methyl-dTTP) labeled with tritium. The amplificate obtained was separated by horizontal agarose-gel electrophoresis in the TAE-buffer at 100 V for 15 min. After the end of electrophoresis, the gel plates were divided into tracks, each of which was cut into 5-mm-long sections. The resulting fragments of the gel were placed into bottles containing 3.0 mL of absolute isopropanol. The bottles were then heated at 80 °C for 2 h. Following extraction of the labeled amplified fragments from the gel, an ordinary toluene scintillator (6.0 mL) was added to the bottles. The results were recorded with a Beta-2 automatic liquid scintillation counter. 

To characterize the degree of damage to DNA, we used the “coefficient of fragmentation” (Cfr) i.e., the ratio of total radioactivity of all tail fractions to that of the head.

### 3.2. Choice of Bioprotectors

Based on the theoretical premises [22,24] and previous successful experiences in attenuating the toxicity of different metallic nanoparticles, including Ag-NPs [43], CuO-NPs [44], and some MeO-NPs combinations including NiO-NPs + Mn_3_O_4_-NPs [23], CuO-NPs + PbO-NPs + ZnO-NPs [26], and Al_2_O_3_-NPs + TiO_2_-NP + SiO_2_-NPs [27]), we chose the following substances for inclusion into what we call a “bioprotective complex” (BPC) to be administered per os along with the 3-month inhalation exposure to NiO-NP:

1. Glutamate is an effective cell membrane stabilizer acting through the intensification of the ATP synthesis under exposure to the damaging action of various cytotoxic particles (e.g., [22,45,46,47]). At the same time, it is one of the precursors of glutathione, which is a powerful cell protector against the oxidative stress. The latter is known to be a key mechanism underlying the cytotoxicity and genotoxicity of virtually all metallic NPs [48]. Alongside these non-specific and almost universal bio-protective effects, glutamate plays a major role in the transmission of excitatory signals in the mammalian central nervous system and is thus involved in most aspects of normal brain functioning. Given this role of glutamate, we assumed that its administration might specifically increase resistance to nickel neurotoxicity.
2. The other two glutathione precursors were chosen—Glycine and cysteine (the latter in a highly active and metabolically well available form of N-acetylcysteine)—given the generally important role played by oxidative stress as a mechanism of metallic NP cytotoxicity and genotoxicity.
3. Other components of the organism’s anti-oxidant system (vitamins A, E, and C, rutin, and selenium) were included as well.
4. ω-3 polyunsaturated fatty acids (PUFA), whose intracellular derivatives are eicosanoids, activate DNA replication and thus play an important part in its repair, as was demonstrated by us earlier under exposure to various genotoxic agents (e.g., [27,30])
5. Iodine supplement was chosen given the complex disturbances of the thyroid function caused by different metallic intoxications.
6. Pectin enterosorbent was also chosen, as it is an agent that hinders both the primary intestinal absorption of metal ions released by MeO-NPs transferred to the GIT from the airways via the throat and the re-absorption of toxic metals excreted into the intestines with bile.

The doses and methods of administration of these bio-protectors were as follows: apple pectin: 1 g/kg body weight (added to the fodder); sodium glutamate: 160 mg per rat (as a 1.5% drink instead of water); glycine: 3 mg per rat (added to the fodder); N-acetylcysteine: 9.3 mg per rat (added to the fodder); vitamin C: 5.65 mg per rat (added to the fodder); rutin: 1.5 mg per rat (added to the fodder); vitamin A: 0.3 mg per rat (added to the fodder); vitamin E: 0.9 mg per rat (added to the fodder); selenium: 1.5 mcg per rat (added to the fodder); potassium iodide: 1.5 mcg per rat (added to the fodder); a commercial fish oil rich in vitamin A and omega-3-rich PUFA: 1 drop per rat (per gavage).

We gave glutamate to rats as a 1.5% solution ad libitum instead of the drinking water. The “Amber Dew” (by Ecco-Plus Ltd., Zhukovskiy, Russia), a fish oil preparation rich in PUFA mainly of the ω-3 group (24%), was administered through gavage at a dose of 1 mL per rat. The apple pectin enterosorbent (by Promavtomatika Ltd., Belgorod, Russia) was added to the rats’ fodder in a quantity corresponding to a dose ca. 1000 mg/kg body mass. The amino acids and vitamins available as tablets were crushed and added to another portion of the fodder in quantities corresponding to the recommended daily intake of these micronutrients by rats (where such recommendations were known only for humans, a recalculation to the rat’s nutritional requirement was made based on the species’ standard metabolism ratio).

Presuming that the standard balanced fodder meets the normal nutritional requirements of rat, we suggested that additional intake of the above-listed bioactive substances would meet the increased needs due to the molecular mechanisms of NiO-NP toxicity. Nevertheless, it had to be checked whether or not such presumed overloading with bioactive substances would evoke any unfavorable effects. That is why in our experiments one group of rats was administered the same BPC but was not exposed to any toxicant.

The statistical significance of the differences between the group arithmetic mean values was estimated using a Student’s *t* test with Bonferroni correction for multiple comparisons. 

## 4. Conclusions

In general, the response of the rat organism to the impact of inhaled nickel oxide nanoparticles corresponds to the patterns that are characteristic of many other previously studied metal and metal-oxide nanoparticle species. The following should be specially noted: (a) typical changes in the cytological and some biochemical characteristics of BALF in response to inhalation of NiO-NPs; (b) various manifestations of systemic toxicity with especially marked damage to the liver and kidneys; (c) some transfer of nanoparticles from the nasal mucous membrane along the olfactory tract, causing damage to the corresponding structures of the brain; (d) a paradoxically mild pulmonary pathology of the pneumoconiotic type, which can be explained by a low chronic retention of nanoparticles in the lungs due to their solubilization in biological milieus; (e) the genotoxic effect on the organism level reliably identified even under low levels of chronic exposure at which the above manifestations of systemic toxicity are rather mild.

However, along with such a generalized toxicological characteristic of metal-oxide nanoparticles, their action quite often involves an important role of harmful effects representative of the toxicity of some specific chemical element [1]. In the case of NiO-NPs, we consider as such the element-specific changes in the red blood indices pointing to the phase-related stimulation of erythropoiesis. We are the first to come across an alternating character of an organism’s response to chronic exposure to nanoparticles and the manifestations of the apparent adaptation to such exposure, judging not only by the hematological but also by other (though far from all) effects.

From the standpoint of preventive and, in particular, regulatory toxicology, we think that the most important conclusion from these studies is that NiO-NP aerosol possesses doubtless, albeit moderate, toxicity even in low concentrations, which in Ni terms either correspond to, or are lower by almost an order of magnitude than, the standards widely adopted for nickel and its compounds in non-nano form. In order to validate such a standard for nickel-oxide nanoparticles, we are currently conducting a similar chronic inhalation experiment involving an even lower concentration of NiO-NPs. However, it is clear that maintaining such low levels of exposure in actual industrial occupational settings could be a major challenge. This makes it especially important to find ways to enhance the resistance of the organism to nano-impacts by means of safe bioprotectors [24]. It is therefore important that in the experiments presented in this paper, we have proved the possibility of such bioprotection (specifically, the significant attenuation of the genotoxic effect of NiO-NPs). 

## Figures and Tables

**Table 1:** Some indices of rat organism status after a short-term inhalation exposure to NiO nanoparticles at a concentration of 1.0 ± 0.1 mg/m^3^—4 h exposure for 5 consecutive days (*x* ± *s.e.*).

Index	24 h after the Fifth Exposure	
	Control	NiO-NP
Body mass before exposure, g	250.0 ± 4.8	242.8 ± 3.3
Body mass after exposure, g	255.63 ± 3.71	247.50 ± 3.56
Body mass gain, %	13.5 ± 5.6	11.4 ± 2.7
Liver mass, g per 100 g body mass	3.12 ± 0.05	3.41 ± 0.07 *
Leukocytes, 10^6^/mL	8.68 ± 0.62	10.94 ± 0.64 *
Erythrocytes, 10^12^/mL	6.92 ± 0.13	7.33 ± 0.14 *
Reticulocytes, ‰	19.01 ± 1.58	46.43 ± 3.14 *
Hemoglobin, g/dL	14.60 ± 0.23	15.44 ± 0.21 *
Hematocrit, %	41.32 ± 0.62	43.32 ± 0.62 *
LDH, U/L	1665.13 ± 273.57	2663.00 ± 360.17 *
MDA in blood serum, μmol/L	5.72 ± 0.37	8.140 ± 1.09 *
SDH activity, number of formazan granules in 50 lymphocytes	800.25 ± 36.65	553.88 ± 10.10 *

Note: * statistically significant difference from the sham-exposed group (*p* < 0.05 by a Student’s *t*-test).

**Table 2:** Number of cells in the bronchoalveolar lavage fluid (BALF) after 4 h inhalation exposures to NiO-NPs at a 1.0 ± 0.1 mg/m^3^ concentration repeated daily for 5 days (*x* ± *s.e.*).

Number of Cells (× 10^6^)				
Group of Rats Exposed to	Total	Neutrophil Leukocytes (NLs)	Alveolar Macrophages (AMs)	NL/AM
**24 h after the Last Exposure**				
**NiO-NP**	6.39 ± 0.68 *	1.37 ± 0.19	5.02 ± 0.61 *	0.29 ± 0.05
**Sham**	1.72 ± 0.34	0.66 ± 0.32	1.06 ± 0.15	0.67 ± 0.39
**21 Days after the Last Exposure**				
**NiO-NP**	3.25 ± 0.24 *	0.33 ± 0.07	2.92 ± 0.27 *	0.12 ± 0.03
**Sham**	1.86 ± 0.13	0.37 ± 0.17	1.50 ± 0.14	0.29 ± 0.14

Note: * statistically significant difference from the sham-exposed group ((*p* < 0.05 by a Student’s *t*-test).

**Table 3:** Some cytological characteristics of different organ imprints from rats sacrificed on the next day after the fifth 4 h inhalation exposure to NiO-NPs at a 1.0 ± 0.1 mg/m^3^ concentration; percentage of total cell count (*x* ± *s.e.*).

Organs and Cells	In Sham-Exposed Rats (Control)	In NiO-HP-Exposed Rats
**Lungs**		
Neutrophils	8.00 ± 0.81	10.29 ± 0.87
Alveolar macrophages	6.25 ± 0.89	6.86 ± 1.30 *
Degeneratively changed alveolar macrophages	6.25 ± 0.81	28.71 ± 3.34 *
Eosinophils	1.75 ± 0.49	10.00 ± 1.31 *
**Liver**		
Degeneratively changed hepatocytes	8.75 ± 0.84	21.86 ± 0.99 *
Neutrophils	7.25 ± 0.66	11.57 ± 0.97 *
Eosinophils	3.50 ± 0.40	7.29 ± 0.68 *
Kupffer cells	3.00 ± 0.68	5.43 ± 0.48 *
**Kidneys**		
Proximal tubule cells	67.75 ± 0.85	51.00 ± 1.60 *
Degeneratively changed cells of proximal tubules	11.33 ± 0.58	22.71 ± 1.64 *
Distal tubule cells	7.67 ± 0.85	10.00 ± 0.72
Degeneratively changed cells of distal tubules	5.67 ± 0.65	8.43 ± 0.65 *
**Spleen**		
Mature lymphocytes, prolymphocytes	85.60 ± 2.29	72.57 ± 1.73 *
Lymphoblasts	0.80 ± 0.20	1.29 ± 0.29
Plasmocytes	1.40 ± 0.24	3.00 ± 0.53 *
Macrophages	2.40 ± 0.24	3.14 ± 0.34
Neutrophils	2.40 ± 0.51	4.43 ± 0.37 *
Eosinophils	4.60 ± 0.68	13.71 ± 0.97 *

Note: * statistically significant difference from the sham-exposed group ((*p* < 0.05 by a Student’s *t*-test).

**Table 4:** Number of cells in the BALF obtained 24 h after the last 4 h inhalation exposure to the NiO-NPs at a 0.23 ± 0.01 mg/m^3^ concentration repeated daily for 3, 6, or 10 months; mean results for at least 12 rats in exposed and sham-exposed groups of each term (*x* ± *s.e.*).

Number of Cells (× 10^6^)				
Group of Rats Exposed to:	Total	Neutrophil Leukocytes (NL)	Alveolar Macrophages (AM)	NL/AM
**3 months**				
**NiO-NP**	21.40 ± 6.01 *	8.40 ± 3.11 *	13.00 ± 3.22 *	0.61 ± 0.11
**Sham**	3.50 ± 0.66	0.43 ± 0.18	3.06 ± 0.69	0.26 ± 0.18
**6 months**				
**NiO-NP**	8.38 ± 0.81 *	1.72 ± 0.26 *	6.67 ± 0.66 *	0.26 ± 0.04 *
**Sham**	2.94 ± 0.53	0.27 ± 0.08	2.78 ± 0.53	0.11 ± 0.04
**10 months**				
**NiO-NP**	9.63 ± 1.15 *	3.74 ± 0.78 *	5.89 ± 0.70 *	0.65 ± 0.12 *
**Sham**	3.44 ± 0.46	0.30 ± 0.08	3.14 ± 0.39	0.09 ± 0.02

Note: * statistically significant difference from the sham-exposed group (*p* < 0.05 by a Student’s *t*-test).

**Table 5:** Some enzymes in the BALF supernatant obtained 24 h after the last 4 h inhalation exposure to NiO-NPs at a 0.23 ± 0.01 mg/m^3^ concentration repeated daily for 3, 6, or 10 months; mean results for at least 12 rats in exposed and sham-exposed groups of each term (*x* ± *s.e.*).

Group of Rats Exposed to:	γ-Gutamyl Transferase	Amylase	Lactate Dehydrogenase	Alkaline Phosphatase	Aspartate Aminotransferase
**3 Months**					
**NiO-NP**	21.76 ± 2.52 *	16.64 ± 2.65 *	218.33 ± 31.9 *	69.61 ± 21.03	23.84 ± 3.61 *
**Sham**	1.04 ± 0.34	2.94 ± 0.41	14.3 ± 2.9	28.49 ± 7.22	4.83 ± 0.42
**6 Months**					
**NiO-NP**	16.56 ± 1.34 *	15.72 ± 2.44 *	360.3 ± 54.6 *	99.48± 18.36 *	37.83± 6.12 *
**Sham**	3.96 ± 1.48	5.11 ± 1.35	16.3 ± 1.1	37.47 ± 6.63	9.39 ± 3.43
**10 Months**					
**NiO-NP**	13.61 ± 2.87 *	9.66 ± 2.49 *	256.00 ± 5.7 *	67.74 ± 21.78 *	37.83± 6.12 *
**Sham**	2.17 ± 0.71	2.03 ± 0.45	19.00 ± 5.7	16.73 ± 5.46	9.39 ± 3.43

Note: * statistically significant difference from the sham-exposed group (*p* < 0.05 by a Student’s *t*-test).

**Table 6:** Some functional indices for rat organism status 24 h after the last 4 h inhalation exposure to NiO-NPs at a 0.23 ± 0.01 mg/m^3^ concentration repeated daily for 3, 6, or 10 months; mean results for at least 12 rats in exposed and sham-exposed groups of each term (*x* ± *s.e.*).

Indices	3 Months		6 Months		10 Months	
	Control	NiO-NP	Control	NiO-NP	Control	NiO-NP
Starting body mass, g	241.2 ± 9.2	230.1 ± 4.9	249.1 ± 42	248.5 ± 5.1	232.9 ± 4.1	230.6 ± 3.6
Final body mass, g	275.4 ± 8.5	262.5 ± 4.8	282.9 ± 6.0	290.3 ± 4.9	287.9 ± 4.3	275.3 ± 7.6
Body mass gain, %	15.2 ± 3.9	14.4 ± 2.3	13.7 ± 2.3	17.4 ± 2.7	25.1 ± 2.8	17.2 ± 1.7 *
Lung mass (dry), g per 100 g body mass	0.15 ± 0.03	0.17 ± 0.01	0.12 ± 0.02	0.15 ± 0.03	0.11 ± 0.01	0.20 ± 0.02 *
Liver mass (wet), g per 100 g body mass	3.24 ± 0.14	3.52 ± 0.13	0.301 ± 0.037	0.250 ± 0.040 *	3.44 ± 0.10	3.41 ± 0.09
Spleen mass (wet), g per 100 g body mass	0.21 ± 0.01	0.22 ± 0.02	0.30 ± 0.06	0.24 ± 0.01	0.21 ± 0.01	0.25 ± 0.02
Kidney mass (wet), g per 100 g body mass	0.63 ± 0.02	0.64 ± 0.02	0.65 ± 0.02	0.69 ± 0.02	0.62 ± 0.02	0.67 ± 0.02 *
Brain mass (wet), g per 100 g body mass	0.70 ± 0.04	0.75 ± 0.02	0.72 ± 0.02	0.71 ± 0.02	0.69 ± 0.02	0.73 ± 0.02
Temporal summation of sub-threshold impulses, sec.	11.02 ± 0.74	10.53 ± 0.47	12.67 ± 0.94	11.31 ± 0.68	11.39 ± 0.82	10.87 ± 0.74
Number of head-dips into holes during 3 min	5.83 ± 1.09	5.58 ± 1.11	6.19 ± 0.84	5.88 ± 0.93	7.00 ± 1.21	8.83 ± 1.13
Number of crossed squares per 3 min	14.25 ± 1.71	12.67 ± 1.54	18.56 ± 2.19	17.31 ± 2.56	12.55 ± 1.54	16.16 ± 1.95
Hemoglobin, g/dL	14.13 ± 0.35	15.82 ± 0.28 *	15.28 ± 0.18	15.11 ± 0.21	15.15 ± 0.38	15.47 ± 0.22
Erythrocytes, 10^12^ cells/L	6.37 ± 0.22	7.08 ± 0.17 *	7.23 ± 0.11	6.94 ± 0.12	6.92 ± 0.24	6.75 ± 0.08
Average volume of red blood cells, µm^3^	58.23 ± 0.65	58.83 ± 0.58	59.90 ± 0.57	62.24 ± 0.57 *	57.85 ± 0.90	59.05 ± 0.44
Hematocrit, %	37.18 ± 1.16	41.56 ± 0.86 *	43.28 ± 0.58	43.18 ± 0.66	39.94 ± 1.22	39.22 ± 0.84
Reticulocytes, ‰	11.92 ± 1.28	16.75 ± 1.39 *	13.73 ± 1.28	20.39 ± 1.56 *	12.55 ± 0.86	15.89 ± 0.62 *
Leukocytes, 10^3^/µL	6.93 ± 0.61	6.00 ± 0.45	5.79 ± 0.42	5.68 ± 0.33	5.62 ± 0.27	6.36 ± 0.38 *
Thrombocytes, 10^3^/µL	0.22 ± 0.02	0.25 ± 0.01	0.26 ± 0.01	0.24 ± 0.01	0.19 ± 0.02	0.22 ± 0.01
Lymphocytes, %	66.67 ± 1.27	69.11 ± 1.05	63.19 ± 1.20	61.20 ± 1.10	53.19 ± 3.78	57.22 ± 0.89
Monocytes, %	6.00 ± 0.41	5.22 ± 0.36	4.69 ± 0.33	4.07 ± 0.34	5.81 ± 0.44	7.67 ± 1.51
Granulocytes, %						
Bilirubin in blood serum, μmol/L	1.09 ± 0.17	1.56 ± 0.14 *	0.96 ± 0.14	1.30 ± 0.09 *	1.14 ± 0.10	1.66 ± 0.23 *
Succinate dehydrogenase (SDH) activity, number of formazan granules per 50 lymphocytes	747.3 ± 9.2	553.3 ± 5.8 *	621.6 ± 10.7	640.7 ± 10.8	619.3 ± 11.1	619.4 ± 6.8
Total protein content of blood serum, g/L	69.37 ± 2.16	69.90 ± 1.65	68.71 ± 1.73	70.43 ± 1.90	72.82 ± 2.37	72.69 ± 2.24
Albumin content of blood serum, g/L	40.78 ± 2.22	39.53 ± 1.16	40.34 ± 1.05	40.50 ± 091	39.63 ± 1.26	39.04 ± 1.52
Globulins of blood serum, g/L	28.59 ± 1.58	30.0 ± 1.04	28.94 ± 1.35	29.92 ± 1.32	33.19 ± 1.41	30.11 ± 2.65
A/G index	1.56 ± 0.24	1.36 ± 0.07	1.43 ± 0.07	1.38 ± 0.05	1.21 ± 0.04	1.17 ± 0.04
AST activity in blood serum, mM/h·L	911.08 ± 616.72	289.88 ± 30.71	193.85 ± 17.24	198.86 ± 11.46	304.02 ± 48.40	279.96 ± 16.65
ALT activity in blood serum, mM/h·L	337.38± 278.35	70.08 ± 10.56	67.01 ± 6.51	61.69 ± 3.52	57.37 ± 4.48	54.42 ± 4.88
De Ritis coefficient	4.89 ± 0.46	4.52 ± 0.32	2.97 ± 0.17	3.34 ± 0.22	5.12 ± 0.39	5.43 ± 0.28
SH-groups in blood serum, µmol/L	10.19 ± 0.53	10.82 ± 0.68	6.67 ± 0.26	6.07 ± 0.95	3.52 ± 0.68	4.31 ± 0.58
Uric acid in blood serum, µmol/L	100.50 ± 7.52	103.7 ± 6.42	102.63 ± 7.66	97.50 ± 7.59	172.75 ± 24.13	146.94 ± 8.33
Urea in blood serum, mmol/L	6.20 ± 2.14	5.04 ± 2.43	2.77 ± 0.15	2.79 ± 0.34	5.52 ± 0.93	4.42 ± 0.46
Activity of γ-glutamintransferase in blood serum, nmol/(s·L)	3.7 ± 1.21	2.87 ± 0.81	2.66 ± 0.80	2.58 ± 1.04	9.89 ± 1.80	9.17 ± 1.37
Creatinine in blood serum, µmol/L	56.43 ± 5.87	48.14 ± 3.26	46.42 ± 2.02	52.61 ± 2.19 *	57.95 ± 3.57	57.54 ± 2.99
Alkaline phosphatase in blood serum, nmol/(s·L)	51.58 ± 6.34	58.58 ± 4.61	135.03 ± 10.63	114.80 ± 13.60	113.63 ± 11.08	90.08 ± 15.05
Catalase in blood serum, µmol/L	0.15 ± 0.03	0.11 ± 0.02	0.54 ± 0.03	0.56 ± 0.03	0.55 ± 0.06	0.60 ± 0.02
Reduced glutathione in the blood hemolysate, µmol/L	31.15 ± 3.63	24.40 ± 3.83	18.36 ± 1.80	18.67 ± 3.96	24.91 ± 1.73	21.12 ± 0.85
Ceruloplasmin in blood serum, mg/%	133.11 ± 12.04	116.83 ± 15.13	67.40 ± 3.70	87.35 ± 10.07	96.49 ± 8.96	120.67 ± 9.22
MDA in blood serum, nmol/L	5.17 ± 0.39	3.97 ± 0.31 *	5.07 ± 0.38	5.74 ± 0.61	5.56 ± 0.72	7.84 ± 0.79 *
Diuresis, mL	31.08 ± 4.72	39.00 ± 2.58	30.58 ± 2.70	26.83 ± 4.54	38.42 ± 3.16	42.19 ± 3.19
Urine relative density	1.01 ± 0.0	1.02 ± 0.0	1.01 ± 0.0	1.02 ± 0.0	1.019 ± 0.0	1.017 ± 0.0
Protein in urine, g/L	144.1 ± 12.9	137.7 ± 7.4	69.8 ± 8.25	108.5 ± 15.94 *	96.4 ± 32.85	181.8 ± 122.39
Urea in urine, mmol/L	95.2 ± 7.8	106.6 ± 14.3	240.3 ± 25.2	235.2 ± 34.7	175.6 ± 9.3	111.2 ± 8.1 *
Uric acid in urine, µmol/L	52.00 ± 11.13	79.73 ± 14.01	181.09 ± 26.8	178.36 ± 29.5	83.25 ± 9.9	57.50 ± 8.1
Creatinine in urine, mmol/L	1.19 ± 0.12	1.01 ± 0.06	1.73 ± 0.12	1.63 ± 0.16	1.27 ± 0.07	1.10 ± 0.09
Endogenous creatinine clearance, ml/min	0.66 ± 3.27	0.82 ± 2.42	1.14 ± 2.2	0.83 ± 2.62	0.84 ± 2.6	0.8 ± 2.51
Genomic DNA fragmentation coefficient in nucleated blood cells	0.4229 ± 0.0008	0.4480 ± 0.0017 *	0.4247 ± 0.0006	0.5332 ± 0.0031 *	0.4244 ± 0.0005	0.5447 ± 0.0036 *

Note: * statistically significant difference from the sham-exposed group (*p* < 0.05 by a Student’s *t*-test with Bonferroni correction).

**Table 7:** Morphometric indices for the state of rat’s liver and spleen after inhalation exposure to NiO-NPs at a 0.23 ± 0.01 mg/m^3^ concentration for 3, 6, or 10 months; mean results for at least 3 rats in exposed and sham-exposed groups of each term, 10 serial microscopic section per an organ in a rat (*x* ± *s.e.*).

Indices	Duration of Exposure					
	3 Months		6 Months		10 Months	
	Sham	NiO-NP	Sham	NiO-NP	Sham	NiO-NP
**Count per 100 Liver Cells**						
Akaryotic hepatocytes	15.7 ± 0.7	17.0 ± 0.7	18.1 ± 0.4	41.8 ± 1.1 *	24.0 ± 0.8	48.5 ± 1.1 *
Binucleated hepatocytes	3.3 ± 0.3	4.9 ± 0.4 *	3.9 ± 0.2	3.1 ± 0.3 *	2.2 ± 0.2	3.7 ± 0.3 *
Kupffer cells	15.2 ± 0.4	16.8 ± 0.4 *	16.5 ± 0.3	18.7 ± 0.4 *	16.8 ± 0.5	19.5 ± 0.4 *
**Spleen Slide Planimetry**						
Red pulp to white pulp ratio	3.8 ± 0.6	3.3 ± 0.5	2.0 ± 0.2	2.2 ± 0.3	2.1 ± 0.3	1.9 ± 0.34
Diameter of the follicle, mcm	12.9 ± 0.4	14.0 ± 0.3	13.2 ± 0.4	15.0 ± 0.5 *	13.0 ± 0.4	14.6 ± 0.5 *

Note: * statistically significant difference from the control group; *p* < 0.05 by a Student’s *t*-test with Bonferroni correction).

**Table 8:** Morphometric indices for the state of rat kidneys after inhalation exposure to NiO-NPs at a 0.23 ± 0.01 mg/m^3^ concentration for 3, 6, or 10 months; mean results for at least 3 rats in exposed and sham-exposed groups of each term, 10 serial microscopic kidneys in a rat (*x* ± *s.e.*).

Indices	Duration of Exposure					
	3 Months		6 Months		10 Months	
	Sham	NiO-NP	Sham	NiO-NP	Sham	NiO-NP
Brush border loss (% lengthwise)	6.7 ± 1.8	15.7 ± 4.0 *	7.9 ± 3.5	17.2 ± 5.1	4.5 ± 2.5	6.2 ± 2.6
Epithelial desquamation (% lengthwise)	1.3 ± 1.1	2.1 ± 1.2	1.1 ± 0.6	1.6 ± 0.8	1.9 ± 0.8	14.1 ± 4.3 *
Glomerular diameter. mcm	35.6 ± 0.7	36.4 ± 0.9	36.6 ± 0.7	37.3 ± 0.8	42.1 ± 0.8	36.0 ± 1.2 *

Note: * statistically significant difference from the control group; *p* < 0.05 by a Student’s *t*-test with Bonferroni correction).

**Table 9:** Some cytological characteristics of different organ imprints in rats sacrificed on the next day after the last 4 h inhalation exposure to NiO-NPs at a 0.23 ± 0.01 mg/m^3^ concentration; percentage of total cell count (*x* ± *s.e.*).

Organs and Cells	In Sham-Exposed Rats (Control)			In NiO-HP-Exposed Rats		
	3 Months	6 Months	10 Months	3 Months	6 Months	10 Months
**Lungs**						
Alveolar macrophages	4.60 ± 0.51	5.43 ± 0.57	4.60 ± 0.51	4.00 ± 0.38	10.29 ± 1.91 *	15.40 ± 0.75 *
Degeneratively changed alveolar macrophages	2.80 ± 1.32	2.29 ± 0.46	3.40 ± 0.81	26.60 ± 3.79 *	28.43 ± 1.84 *	34.00 ± 1.38 *
Neutrophils	20.00 ± 0.55	7.86 ± 0.50	4.40 ± 0.51	14.40 ± 2.46 *	3.14 ± 0.46 *	2.40 ± 0.24 *
Eosinophils	4.00 ± 0.66	2.00 ± 0.20	0.80 ± 0.20	4.20 ± 0.86	4.00 ± 0.44 *	0.40 ± 0.24
**Liver**						
Degeneratively changed hepatocytes	8.75 ± 0.84	3.29 ± 0.42	5.33 ± 0.42	21.86 ± 0.99 *	11.43 ± 0.87 *	8.17 ± 0.48 *
Neutrophils	7.20 ± 0.66	3.71 ± 0.68	5.83 ± 0.40	11.57 ± 0.97 *	8.14 ± 0.51 *	11.17 ± 0.95 *
Eosinophils	3.50 ± 0.40	0.71 ± 0.18	3.50 ± 0.67	7.29 ± 0.68 *	4.29 ± 0.61 *	3.00 ± 0.37
Kupffer cells	3.00 ± 0.68	1.29 ± 0.18	3.17 ± 0.60	5.43 ± 0.48 *	4.14 ± 0.91 *	4.67 ± 0.49
**Kidneys**						
Cells of proximal tubules	67.75 ± 0.85	67.86 ± 2.01	71.50 ± 0.85	51.00 ± 1.60 *	61.14 ± 1.44 *	57.71 ± 1.38 *
Degenerative cells of proximal tubules	11.33 ± 0.58	5.29 ± 0.29	5.50 ± 0.45	22.71 ± 1.64 *	12.00 ± 0.82 *	13.00 ± 0.76 *
Cells of distal tubules	7.67 ± 0.85	10.00 ± 0.93	8.83 ± 0.76	10.00 ± 0.72	5.29 ± 0.61*	6.43 ± 0.48 *
Degenerative cells of distal tubules	5.67 ± 0.65	4.71 ± 0.42	4.33 ± 0.22	8.43 ± 0.65 *	9.43 ± 0.65 *	9.43 ± 0.53 *
**Spleen**						
Mature lymphocytes, prolymphocytes	85.60 ± 2.29	90.00 ± 0.69	90.50 ± 0.29	72.57 ± 1.73 *	82.43 ± 1.49 *	79.00 ± 1.95 *
Lymphoblasts	0.80 ± 0.20	1.29 ± 0.18	1.17 ± 0.18	1.29 ± 0.29	0.86 ± 0.14	0.71 ± 0.18 *
Plasmocytes	1.40 ± 0.24	2.57 ± 0.72	2.33 ± 0.85	3.00 ± 0.53 *	3.57 ± 0.95	5.00 ± 0.79 *
Macrophages	2.40 ± 0.24	1.43 ± 0.30	1.17 ± 0.18	3.14 ± 0.34	2.14 ± 0.40	2.71 ± 0.42 *
Neutrophils	2.40 ± 0.51	2.57 ± 0.48	2.67 ± 0.50	4.43 ± 0.37 *	5.71 ± 0.47 *	7.00 ± 0.53 *
Eosinophils	4.60 ± 0.68	2.57 ± 0.43	2.67 ± 0.53	13.71 ± 0.97 *	4.71 ± 0.52 *	4.86 ± 0.74 *

Note: * statistically significant difference from the control (sham exposed) group ((*p* < 0.05 by a Student’s *t*-test).
